# The Many Faces of NLRs in Macrophage Biology∗

**DOI:** 10.1016/j.jacbts.2023.02.004

**Published:** 2023-05-22

**Authors:** Nikolaos G. Frangogiannis

**Affiliations:** Wilf Family Cardiovascular Research Institute, Department of Medicine (Cardiology), Department of Microbiology and Immunology, Albert Einstein College of Medicine, Bronx, New York, USA

**Keywords:** cardiac remodeling, heart failure, immunomodulatory therapy, macrophages, NOD-like receptor

**Figure:**
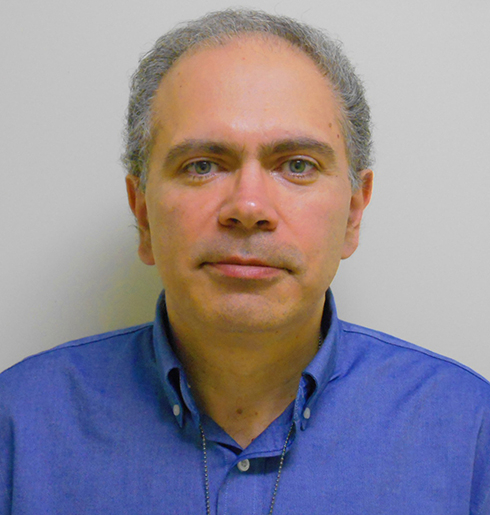

Left ventricular pressure overload plays an important role in the pathogenesis of heart failure in both heart failure with reduced ejection fraction, and heart failure with preserved ejection fraction. In patients with hypertension, and in individuals with obstruction of left ventricular outflow due to anatomical stenotic lesions, the left ventricle is subjected to a pressure load, which triggers a constellation of adaptive and maladaptive cellular changes that ultimately lead to functional perturbations, culminating in the development of heart failure. The cell biological alterations in the pressure-overloaded heart involve phenotypic changes in several different cell types, including cardiomyocytes, fibroblasts, vascular cells, and immune cells.

Cardiac macrophages have been implicated as critical effector cells in the regulation of both injurious and adaptive cellular responses in the pressure-overloaded myocardium, exerting a broad range of functions.^1^, ^2^, ^3^ The functional diversity of macrophages in the pressure-overloaded heart reflects, at least in part, the phenotypic heterogeneity of macrophage populations. In mouse models of left ventricular pressure overload, macrophages expressing the chemokine receptor CCR2 secrete proinflammatory mediators and may be implicated in the pathogenesis of fibrosis and dysfunction.^4^ In contrast, a subpopulation of macrophages expressing insulin growth factor–1 mediates the adaptive hypertrophic response, necessary for the heart to withstand hemodynamic stress.^5^ Other subpopulations of macrophages infiltrating the pressure-overloaded myocardium have been suggested to stimulate angiogenesis and inhibit fibrosis.^2^ In addition to the effects of different subpopulations, the diverse functions of macrophages may also be explained by their remarkable phenotypic plasticity. In the dynamic environment of the pressure-overloaded heart, cardiac macrophages can sense mechanical stress,^6^ changes in the extracellular matrix network,^7^ and the presence of secreted cytokines and growth factors and respond by altering their phenotype and their functional profile. The molecular mechanisms that mediate macrophage transformations in response to the microenvironmental changes in remodeling hearts remain poorly understood.

In this issue of *JACC: Basic to Translational Science*, Yu et al^8^ identify a novel molecular pathway involved in anti-inflammatory transition of myocardial macrophages following pressure overload. The investigators report that induction of the NOD-like receptor NLRC5 in macrophages infiltrating the pressure-overloaded myocardium suppresses myocardial inflammation and protects the heart from adverse remodeling and dysfunction. The anti-inflammatory actions of NLRC5 in macrophages are mediated through binding to the chaperone HSPA8. The NLRC5/HSPA8 interaction inhibits phosphorylation of the IKKb subunit of the IKK complex, thus attenuating NF-κB activation and proinflammatory cytokine synthesis. These findings suggest a novel mechanism of anti-inflammatory macrophage activation in the pressure-overloaded heart, highlighting the significance of these versatile cells in regulation of myocardial inflammation. Moreover, the observations contribute to our understanding of the biological functions of NLRs, which extend beyond the well-characterized proinflammatory actions of the inflammasome-forming members of the family.

## The Many Faces of the NLRs

NLRs are a family of structurally related pattern recognition receptors involved in recognition of microbial pathogens, or danger signals, and subsequent activation of a proinflammatory response. NLR proteins can be further classified into subfamilies on the basis of the composition of their N terminus, which can contain acid transactivation, pyrin, CARD, or BID domains. Several of the pyrin domain-containing NLRs (NLRPs) are involved in the assembly of inflammasome platforms, thus mediating downstream activation of proinflammatory signaling cascades. NLRP3, the best characterized member of the NLRP subfamily, is a central component of the inflammasome platform and is critically involved in generation of active IL-1β in the pressure-overloaded myocardium.^9^ In contrast, limited information is available on the role of the CARD-containing NLRs (NLRCs). NLRC5, the largest protein in the NLR family, is expressed predominantly by hematopoietic cells and has been suggested to play a role in antigen presentation by stimulating MHC class I gene synthesis. Although several in vitro studies and experiments in global loss-of-function models have suggested effects of NLRC5 beyond the MHC I pathway, these functions remain controversial.^10^

## The Role of NLRC5 in the Regulation of Macrophage Phenotype

**Figure 1:**
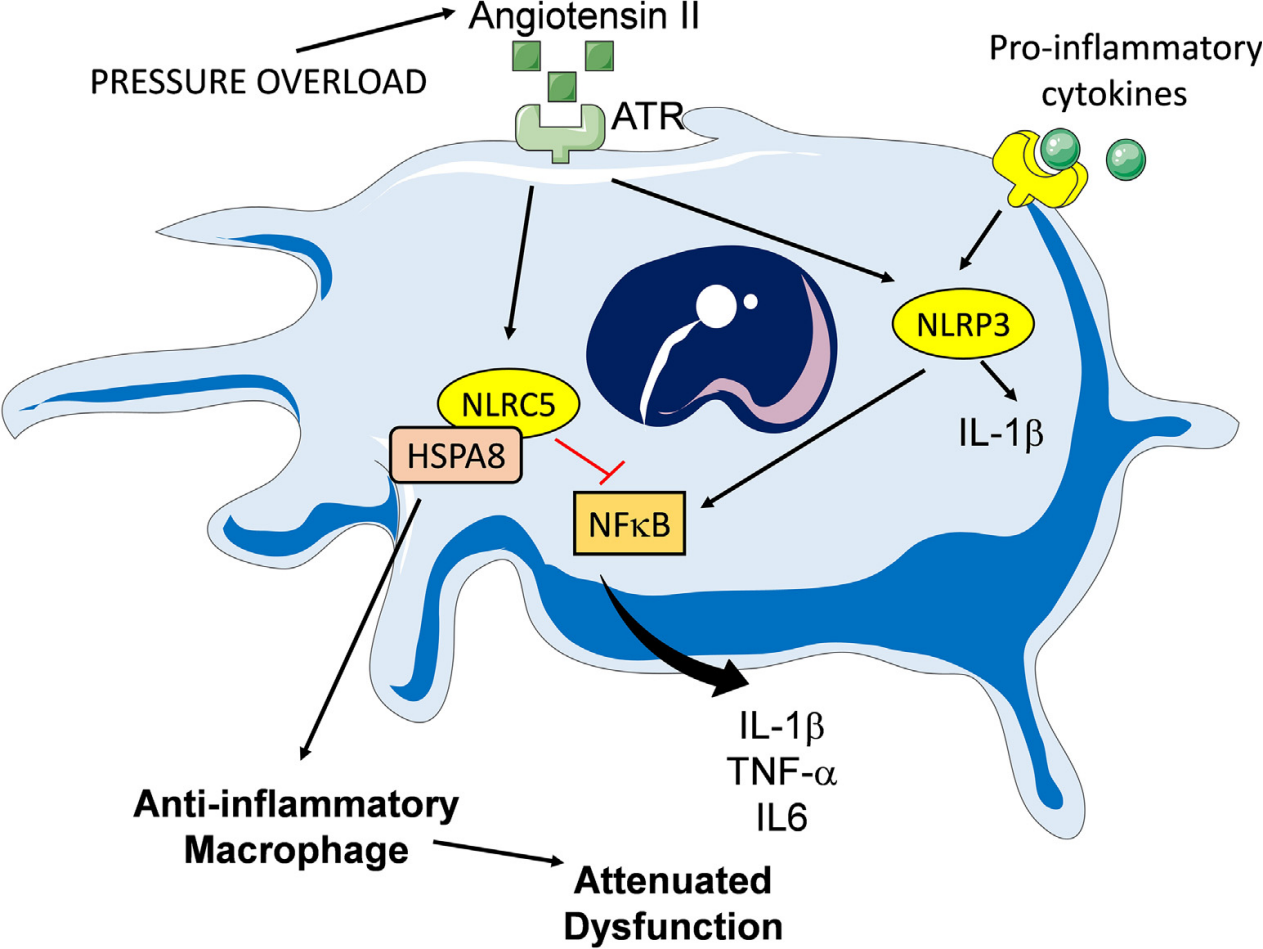
NLRs Regulate Macrophage Phenotype and Function in the Pressure-Overloaded Myocardium Proinflammatory mediators and angiotensin II are known to activate the NLRP3 inflammasome, inducing synthesis of cytokines (such as IL-1β, IL-6, and TNF-α) that promote adverse remodeling and dysfunction. The study by Yu et al^8^ demonstrates that left ventricular pressure overload is also associated with induction of NLRC5, an NLR with anti-inflammatory actions. NLRC5 may be induced by neurohumoral mediators (such as angiotensin II) and inhibits NF-κB signaling and downstream cytokine expression through binding with the chaperone HSPA8. Thus, in addition to its proinflammatory actions, angiotensin II may also promote late activation of an anti-inflammatory program that restrains NF-κB-driven inflammation. ATR = angiotensin receptor; IL = interleukin; NLR = NOD-like receptor; NLRC5 = NOD-like receptor family caspase recruitment domain family domain containing 5; TNF = tumor necrosis factor.

In vitro studies and investigations using mice with global loss of NLRC5 have produced conflicting results, suggesting both pro- and anti-inflammatory actions on macrophages.^10^ The present study provides the first in vivo evidence supporting an anti-inflammatory role for NLRC5 in macrophages, supported by experiments using a myeloid cell–specific loss-of-function approach. The anti-inflammatory effects of NLRC5 were attributed to attenuation of NF-κB activity, mediated through an interaction with HSPA8 (Figure 1). However, whether these effects of NLRC5 are generalizable in other tissues and pathophysiologic conditions associated with macrophage activation is unknown. Macrophages are highly heterogeneous and may exhibit distinct responses to NLR activation depending on their baseline phenotype. Moreover, different pathologic conditions are associated with distinct patterns of macrophage activation that may greatly affect the consequences of NLRC5 activation. Studies using robust macrophage-specific deletion strategies in various inflammatory pathologies are needed to establish the involvement of NLRC5 in negative regulation of inflammation.

## The Role of Neurohumoral Mediators in NLR Activation

Induction of NLRC5 in immune cells typically involves stimulation with Toll-like receptor ligands (such as lipopolysaccharide) or inflammatory cytokines (such as IFN-g). The present study proposes a new mechanism that may link mechanical activation with induction of NLRC5 in macrophages infiltrating the pressure-overloaded myocardium. Angiotensin II, a neurohumoral mediator with a critical role in cardiac remodeling, was found to stimulate a late up-regulation of NLRC5 expression in macrophages. Angiotensin II is known to activate the NLRP3 inflammasome in macrophages^11^ and in other cell types; this effect may account, at least in part, for its proinflammatory actions. The late induction of the anti-inflammatory NLR, NLRC5, noted by the investigators may suggest an endogenous suppressive mechanism triggered by angiotensin II to restrain its proinflammatory actions. The late time course of NLRC5 up-regulation indicates that these suppressive effects may not be mediated directly by angiotensin and angiotensin receptor signaling but may reflect secondary actions of an angiotensin-induced proinflammatory signal.

## Manipulating Macrophages to Attenuate Dysfunction of the Failing Heart

Considering their prominent role in cardiac remodeling and dysfunction, macrophages may be promising therapeutic targets in chronic heart failure. Manipulation of macrophages to achieve temporally restricted suppression of proinflammatory mediators may attenuate cytokine-induced ventricular dysfunction. Inhibition of the fibrogenic properties of macrophages may reduce ventricular stiffness, improving diastolic function. Induction of an angiogenic macrophage profile may increase perfusion. Targeting NLRs may be a highly effective approach in the modulation of macrophage profile in heart failure and in other diseases associated with prominent inflammatory responses. Currently, pharmacologic approaches have focused almost exclusively on inhibition of the NLRP3 inflammasome. Although the emphasis on the development of NLRP3-targeting therapeutics is well justified, the present investigation shows that other members of the NLR family may also hold promise as therapeutic targets.

## Funding Support and Author Disclosures

Dr Frangogiannis’s laboratory is supported by National Institutes of Health grants R01 HL76246, R01 HL85440, and R01 HL149407 and by U.S. Department of Defense grants PR181464 and PR211352.
